# The Scolioscope: a home detection tool for measuring axial trunk rotation in scoliosis—a validation study

**DOI:** 10.1007/s43390-025-01174-0

**Published:** 2025-09-08

**Authors:** Julia H. E. Holleman, Hanneke M. van West, Thomas Q. M. Vu, Max Reijman, Joost P. H. J. Rutges

**Affiliations:** Department of Orthopedics and Sports Medicine, Erasmus MC, University Medical Center Rotterdam, Dr Molewaterplein 40, 3015 GD Rotterdam, The Netherlands

**Keywords:** Scoliosis, Adolescent, Screening, Self-test, Adam forward bending test, Scolioscope

## Abstract

**Purpose:**

Screening for adolescent idiopathic scoliosis (AIS) using the Adam Forward Bending Test (AFBT) remains controversial, resulting in the discontinuation of scoliosis screening in the Netherlands. This study aims to validate the Scolioscope, a simplified version of the Scoliometer, for detecting scoliosis in a home setting.

**Methods:**

A validation study was conducted at the orthopedic outpatient clinic of Erasmus Medical Center Sophia Children's Hospital in Rotterdam, the Netherlands. Patients aged 9–18 years with or without AIS and capable of performing the AFBT were included. The Scolioscope measurement of the parents was compared with the Scoliometer measured by an orthopedic surgeon. After unsatisfactory results with the initial Scolioscope version, a revised version was developed and tested.

**Results:**

Among 100 patients included in the study, 79 had scoliosis. The revised version of the Scolioscope demonstrated a positive predictive value of 97%, a negative predictive value of 89%, sensitivity of 94%, and specificity of 94%. Parental measurements showed no variation, with an intra-observer reliability kappa value of 1.

**Conclusion:**

The Scolioscope demonstrates high diagnostic accuracy and precision, making it suitable for use in at-home scoliosis screening programs.

## Introduction

If and how to screen for adolescent idiopathic scoliosis (AIS) has been a matter of debate for several decades. Several studies on scoliosis screening show conflicting outcomes [1–7]. Early detection allows nonoperative treatment options to be installed in time [8]. Home detection creates awareness of scoliosis, empowering caregivers to take appropriate actions. Disadvantages of screening are potential stress and anxiety associated with the possible diagnosis of scoliosis. Other disadvantages are false positive results that can lead to overtreatment and false negative results leading to a false sense of security.

In the Netherlands, scoliosis screening was abandoned in 2014 after a report by the Dutch Organization for Applied Scientific Research (TNO) [9]. The report stated insufficient evidence to support the effectiveness of screening. However, abandoning screening may contribute to delayed detection. A Danish study found significantly larger curve magnitudes at referral in non-screened populations compared to screened ones (22% versus 8% with a Cobb angle > 40 degrees, *p* < 0.001) [6]. This suggests that non-screened populations result in later detection of scoliosis, leading to more severe curves, potentially requiring more surgical interventions. Surgery should nevertheless be considered with caution as it can be accompanied by major complications [10, 11]. Conservative treatment should be initiated in mild to moderate scoliosis (Cobb angle < 40 degrees) before skeletal maturity to prevent progression. Reports indicate that the efficacy of bracing may be as high as 74% to 81% in halting the progression of AIS [12]. Hence, it is crucial to detect the scoliosis at an early stage.

This resulted in a reevaluation of screening approaches, addressing concerns such as cost, personnel training, and logistical requirements. To revive the discussion on screening for scoliosis, scientific proof of its effectiveness needs to be obtained. The project team focuses on designing an effective, future-proof screening program, and determining the appropriate tools for implementation. Our project consists of the evaluation of this innovative at-home screening tool empowering parents/caregivers to check the spine of their children.

The Scoliometer is the gold standard for detecting scoliosis, using the Adam Forward bending test (AFBT). A reading greater than 5 to 7 degrees generally indicates a rotational deformity significant enough to warrant referral to a physician [12]. This can accurately determine which patients require further evaluation or referral [13]. This study aims to validate an at-home screening tool for the detection of scoliosis. More precisely, it will evaluate the accuracy and precision of the Scolioscope, a simplified version of the Scoliometer. The Scolioscope is designed to detect AIS when used during the AFBT. It has been developed to be used by nonhealthcare providers and is therefore intended for self-detection at home by parents/caretakers. Once this tool is validated, it can be tested in a home detection program. This validation study is the first step in improving methods for early scoliosis detection.

## Methods

### Study design

Patients were evaluated during one visit at the outpatient clinic, in which the order of AFBT measurements was randomized. A Medical Centre ethics committee approved the research protocol (MEC-2020-0597). Written informed consent was obtained from the parents/legal guardians and patients. Children were informed in an age-appropriate manner and participated voluntarily. Their written assent was also obtained.

A second period of enrollment was installed after the initial version of the Scolioscope yielded unsatisfactory results, prompting the development of a second version of the Scolioscope and consequently a second validation period.

This manuscript is written according to the Reporting of Diagnostic Accuracy Studies (STARD) [14].

### Participants

Consecutive patients were recruited between January 2021 and March 2021, and between August 2022 and November 2022. Patients were recruited from the Erasmus Medical Center Sophia Children's Hospital in Rotterdam, the Netherlands visiting the scoliosis outpatient clinic. Both children with and without scoliosis were eligible for inclusion in the study. The inclusion criteria encompassed age between 9 and 18 years, and the ability to perform the AFBT. Patients with previous limb or spinal surgery, current brace treatment, other diseases affecting posture or trunk shape, leg length discrepancy > 2 cm, or absence of a parent or caretaker during the appointment were excluded from the study.

### The instrument

The Scolioscope measures axial trunk rotation (ATR) reflecting the main study parameter. Measurement of the ATR is possible with the AFBT. To perform the AFBT correctly, the patients must bend forward with stretched knees until their trunk is parallel to the ground, keeping the palms of their hands together while their arms are hanging down. To discover the biggest rotation of the Gibbus deformity, the Scoliometer and Scolioscope were guided down along the spine in the horizontal plane of the vertebral column [15]. The ATR is the angle between a horizontal reference line and the plane across the back at the greatest elevation of rib prominence or lumbar prominence [6].

**Fig. 1:**
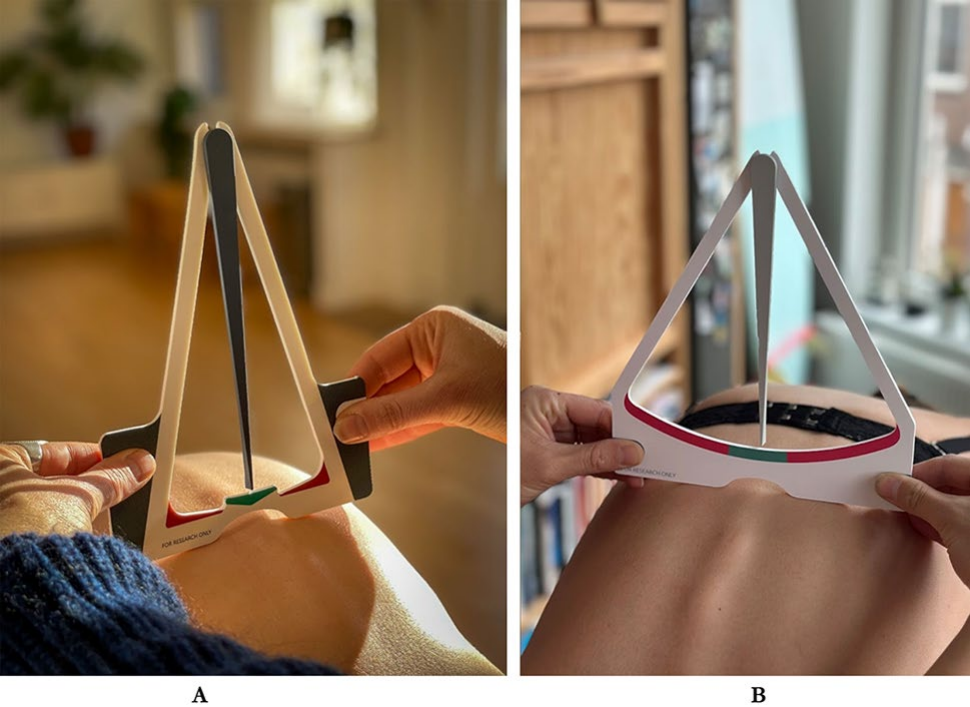
**A** Scolioscope version 1 (with a cut-off point of 5 degrees). **B** Scolioscope version 2 (with a cut-off point of 6 degrees)

The Scolioscope is an adapted version of the Scoliometer where the inclinometer is replaced by a gravity-operated sling. The Scolioscope is cheaper, namely less than €0.50 when produced at large scale, envelope-compatible, and offers a more economical and easily distributable alternative to the bulkier and costlier Scoliometer. The initial version of the Scolioscope has a cut-off point of 5 degrees, above which the child is suspected to have scoliosis (Fig. 1A). Based on the unsatisfactory results of this version, the Scolioscope was adapted.

The revised version of the Scolioscope (Fig. 1B) has a wider and different shape, designed to enhance usability for parents/caretakers. Moreover, the cut-off value was changed to 6 degrees and determined the accuracy and precision of this new version.

### Study procedures

Baseline characteristics (age, sex, body mass index (BMI)) were collected, together with the Cobb angle, determined on a standing posteroanterior (PA) radiograph. AFBT measurements with the Scolioscope were performed twice by a parent or caretaker of the child. The measurement with the Scoliometer (gold standard) was performed once by an experienced orthopedic spine surgeon. The order of the person (parent/caretaker versus orthopedic surgeon) who performed the measurement was randomized using block randomization with blocks of 2–6 and implemented using Microsoft Access. The measurements were done blinded, by measuring with the instrument facing away from the person performing the measurement. An independent researcher reported the outcome of the measurement.

Besides the ATR measurements, the usability of the Scolioscope was evaluated using the System Usability Score (SUS) questionnaire [16]. The SUS provides an accurate tool for measuring usability. It consists of a 10-item questionnaire with five response options for respondents: from strongly agree to strongly disagree. The SUS yields scores in the range of 0 to 100, with a commonly accepted threshold for a high score being above 70 [17].

### Statistical analysis

#### Accuracy

Accuracy was assessed by using the first parental measurement to calculate the positive (PPV), and negative predictive value (NPV), sensitivity (sens), and specificity (spec) of the Scolioscope compared to the Scoliometer. The Scoliometer was used as current practice to assess the axial trunk rotation. The Scolioscope was additionally compared to the Cobb angle, defining scoliosis as 10 degrees or more [18]. Good accuracy was defined as a PPV of > 80%, and a NPV of > 90% [19].

#### Precision

Intra-observer reproducibility of the Scolioscope was assessed by using the kappa-value statistic (κ). This was assessed by comparing the two measurements of the parents / caregivers. A good precision was determined by a kappa value of ≥ 0.7.

Data were analyzed using IBM SPSS Statistics, version 26.0.0.0 for Windows (Armonk, NY:IBM Corp).

#### Usability

Usability of the Scolioscope was assessed using the System Usability Scale (SUS). The SUS score was computed using a standardized formula based on respondents’ ratings, providing a concise measure of usability [17]. Testing the usability is necessary to ameliorate the Scolioscope, where appropriate, to meet users' needs.

#### Sample size calculation

The required sample size was based on:

1. Sample size calculation with an expected ICC value of 0.8 with 2 repeated measurements requiring 50 inclusions. 2. The Cosmin criteria requiring 50–99 patients to meet a good qualified methodological study [20].

## Results

### Version 1

#### Population

Fifty patients met the inclusion criteria and were included, of which 40 patients (80%) had scoliosis as confirmed by the Cobb angle (see Table 1).Query

**Table 1:** Baseline characteristics

	Version 1 (*n* = 50)	Version 2 (*n* = 50)
Sex, female *n* (%)	35 (70)	37 (74)
Age, years	14 (1.8, 10–17)	14 (1.8, 11–17)
BMI, kg/m^2^	20 (3.5, 14–29)	19 (2.8, 8–26)
Cobb angle*, degrees	24 (14, 0–50)	27 (20, 0–70)

Data is presented as mean and standard deviation and min–max (range) between parentheses, or reported otherwise

^*^Cobb angle was determined on a standing posteroanterior (PA) radiograph

#### Accuracy

The Scolioscope version 1 had a PPV of 96%, a NPV of 64%, a sensitivity of 77% and a specificity of 93% compared to the Scoliometer (Table 2). Compared to the Cobb angle, this Scolioscope had a PPV of 96%, a NPV of 46%, a sensitivity of 69% and a specificity of 91% (Table 3).

**Table 2:** Accuracy of both Scolioscope versions compared to the scoliometer

		Version 1	Version 2
Accuracy	PPV, %	96 (95% CI 85–99)	97 (95% CI 87–99)
	NPV, %	64 (95% CI 43–81)	89 (95% CI 70–98)
	Sensitivity, %	77 (95% CI 62–89)	94 (95% CI 80–98)
	Specificity, %	93 (95% CI 74–97)	94 (95% CI 76–99)

*PPV* positive predictive value, *NPV* negative predictive value

Positive result defined as ATR ≥ 5° (Version 1), ATR ≥ 6° (Version 2), or ATR ≥ 7° (Scoliometer)

**Table 3:** Accuracy of both Scolioscope versions compared to the Cobb angle

		Version 1	Version 2
Accuracy	PPV, %	96% (95% CI 85–99)	97% (95% CI 87–.998)
	NPV, %	46% (95% CI 26–66)	56% (95% CI 33–77)
	Sensitivity, %	69% (95% CI 54–82)	80% (95% CI 65–90)
	Specificity, %	91% (95% CI 66–96)	91% (95% CI 66–96)

*PPV* positive predictive value, *NPV* negative predictive value

Positive result defined as ATR ≥ 5° (Version 1), ATR ≥ 6° (Version 2), or ≥ 10° (Cobb angle)

#### Precision

The intra-observer reliability of version 1 had a Kappa value of 0.80.

#### Usability

The mean score of all 50 questionnaires was 78.5 (SD, 14.3; min. to max. 45–100), which falls at the 80–84th percentile and corresponds to a B + grade. Several patients reported that the Scolioscope was not easily usable due to friction of the needle. Sliding it down the spine while holding it vertically was also an issue, according to multiple users. A few patients commented on the sharp-edged side of the Scolioscope.

#### Adaption of version 1

**Fig. 2:**
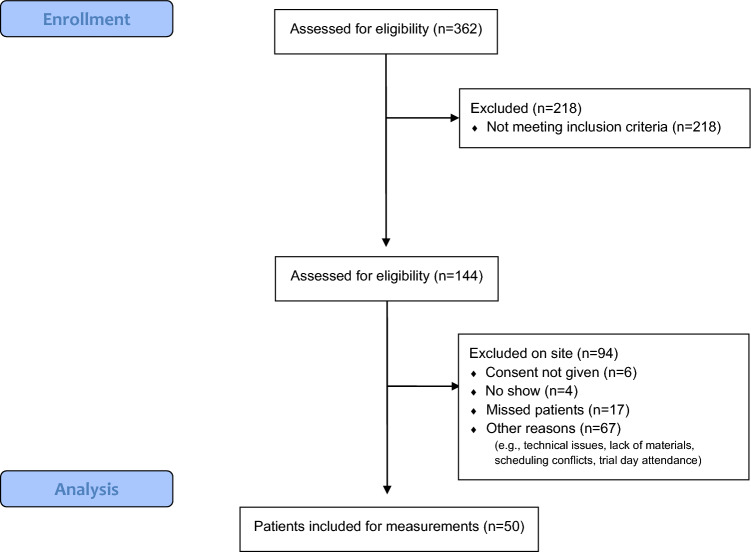
CONSORT flow diagram inclusion Jan–Mar 2021

**Fig. 3:**
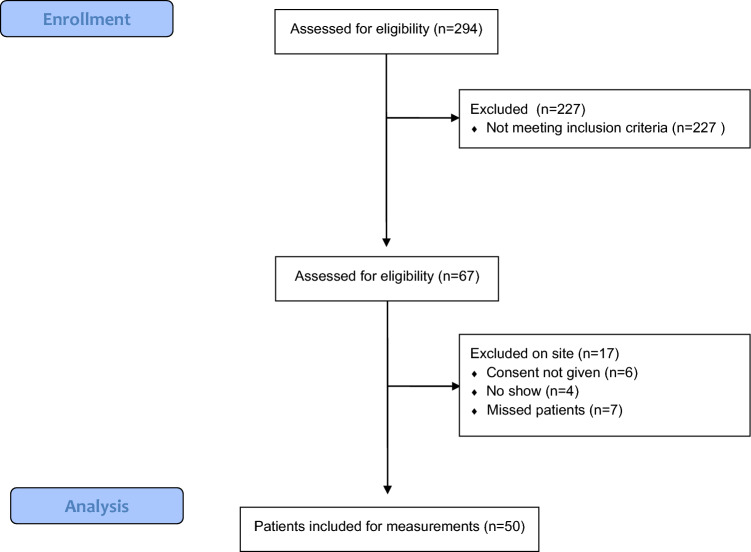
CONSORT flow diagram inclusion Aug–Nov 2022

After the initial Scolioscope (Fig. 1A) yielded unsatisfactory results, a second version was developed. In the revised Scolioscope (Fig. 1B), the test positivity cut-off value was raised to 6, enhancing scoliosis detection precision. Findings from the initial version of the SUS were also incorporated. The device was redesigned with a wider and more user-friendly shape, prioritizing ease of use for parents and caretakers (see Figs. 2, 3).

### Version 2

A second period of enrollment was installed and consequently a second validation period.

#### Population

During the period from August to November 2022, an additional 50 patients were enrolled, out of which 39 (78%) were diagnosed with scoliosis.

#### Accuracy

The Scolioscope version 2 had a PPV of 97%, a NPV of 89%, a sensitivity of 94% and a specificity of 94% compared to the Scoliometer (Table 2*)*. Compared to the Cobb angle, this Scolioscope had a PPV of 97%, a NPV of 56%, a sensitivity of 80% and a specificity of 91% (Table 3).

#### Precision

There was no variation between the measurements performed by the parents. Therefore, the intra-observer reliability had a kappa value of 1.

## Discussion

Our study evaluated the Scolioscope’s accuracy and precision by comparing it to the gold standard device for clinically assessing scoliosis: the Scoliometer. The first version of the Scolioscope demonstrated good precision but lacked accuracy. The revised Scolioscope version results show high precision and good accuracy. These results suggest that the Scolioscope can be used in untrained hands.

Various instruments for measuring ATR in scoliosis screening have been studied. To prevent any confusion with prior work, we wish to clearly differentiate our approach from the study by Krekoukias et al. [21]. It evaluated a digital device also named the Scolioscope, but focused on technical accuracy (using a sine bar) and intra-rater reliability (ICC) of a digital device. In contrast, our study validates a fundamentally different tool. Mobile applications on a smartphone have been explored as an alternative to the Scoliometer to replicate its functionality [22–27]. However, these applications are primarily available for iOS, limiting their accessibility. Additionally, their accuracy requires further validation, as some studies were conducted in non-clinical settings. Measuring ATR with mobile applications can be affected by phone quality and user’s technique. Moreover, while apps might be accessible, they often require technical knowledge and familiarity with smartphones. A physical instrument with clear instructions can be simpler for parents to use consistently and correctly. The Scolioscope is designed specifically for measuring the ATR and provides clear cutoff values, offering straightforward binary results. Wei et al. [28] assessed the reliability and validity of a handheld scanner (SpineScan3D) with built-in electronic sensors for automatically detecting surface profiles three-dimensionally. This instrument is relatively expensive at $64. With a sensitivity ranging from 72 to 74% and specificity ranging from 62 to 74%, it does not offer superior diagnostic accuracy compared to the Scolioscope. Romano et al. [29] evaluated the torsion bottle for home use by parents and found it effective for basic scoliosis screening. However, it lacks a scale or clear cutoff values, and the bottle shapes vary by country, limiting its universality. In conclusion, while mobile applications and various instruments have been explored for measuring ATR in scoliosis screening, the Scolioscope stands out due to its user-friendly design and precise results. It offers practical advantages for home use, provides clear cutoff values and binary results, making it an accurate tool for parents/caregivers to screen scoliosis.

Our study has some limitations that affect the generalizability of our findings. First, referral bias may have occurred due to the recruitment of patients from an orthopedic outpatient clinic [30]. The study included children both with and without scoliosis; however, 79 out of 100 have scoliosis. This leads to an overrepresentation of more severe or specialized cases than would have been found in the general population. As a result, our findings cannot be generalized to the open populations with different demographic or clinical characteristics. Another limitation in scoliosis screening is the diagnostic value of the Scoliometer. Although the Scoliometer is the current practice for measuring ATR, it is not the current practice for detecting scoliosis, which remains the X-ray. An ATR measured 5 degrees or higher does correspond to a Cobb angle of over 20 degrees [31], which is clinically relevant since bracing treatment typically starts at 25 degrees [32]. The sensitivity of the Scoliometer varies from 0.62 to 1.0, depending on the chosen cutoff point. Typically, a 7° ATR cutoff is used, with sensitivity ranging from 0.62 to 0.86 and specificity from 0.67 to 0.75, leading to several false positive or negative cases [33–35]. The Scolioscope was also compared to the Cobb angle, with version 2 showing a sensitivity of 80% (95CI 65–90) and specificity of 91% (95%CI 66–96). Though these results are not highly impressive, the AFBT with ATR measurement remains the most accurate, precise, and simple test for detecting scoliosis without radiography [36]. A cutoff value of 6° was selected for the ATR measurement. The 5° threshold yielded unsatisfactory results, while the 7° threshold risked missing initial cases in the first screening step. Those identified at 6° or more will be further evaluated in subsequent steps. The Scolioscope is cut from 2 mm foamed PVC sheet (Forex®), selected for its rigidity, low weight, and cleanability. It includes a polyethylene pointer mounted on a miniature ball bearing. The instrument withstood > 100 flex cycles and can be disinfected with alcohol wipes without damage. A known limitation is that the firm edges may feel uncomfortable if pressed too hard against skin; this may serve as tactile feedback to encourage light contact. If needed, mass production allows for softening the edges. The simplicity of the device makes it non-patentable from a utility-patent perspective. However, to discourage direct visual imitation, its design has been registered under the EU Registered Community Design (RCD) system. This form of protection prevents copying of the overall appearance, while still allowing others to produce functionally similar tools as long as their design is recognizably different.

Although artificial intelligence (AI) offers promising avenues for scoliosis detection, we deliberately chose a low-tech, physical approach for the Scolioscope. Our earlier experience with a smartphone inclinometer app revealed practical barriers—including installation issues, poor timing of notifications, and device availability—that undermined real-world use. In contrast, a tangible tool placed in the wardrobe serves as a behavioral cue linked to growth, improving adherence. The Scolioscope also includes foot and hand markers that standardize posture and reduce false positives, which is challenging to achieve with smartphone-based assessments. Moreover, physical tools avoid privacy concerns and regulatory burdens associated with AI apps, enabling equitable, low-cost, and scalable home screening.

To further evaluate the effectiveness of the Scolioscope, the next step involves testing its usability in a home setting and assessing its impact on hospital referrals. Pragmatic trials should be conducted to assess the benefits, feasibility, and usability of the Scolioscope under real daily conditions to make them similar to the general population. Moreover, referral to an experienced trained practitioner for an initial follow-up after a positive Scolioscope measurement at home would ensure that a physical examination has been performed appropriately.

Once established, the Scolioscope can streamline scoliosis screening, offering time and cost savings to healthcare providers across specialties, including orthopedic surgeons, nurses, physician assistants, and primary care physicians. This study demonstrates that the Scolioscope is an alternative to standard measurement techniques when used appropriately. In the existing clinical pathway, these results could be positioned in studying the possibility of an at-home screening program for scoliosis detection. In conclusion, the Scolioscope, with its high diagnostic accuracy and precision, enables regular at-home checks during growth spurts, facilitating early detection of AIS potentially as part of a home screening program.

## Data Availability

Individual de-identified participant data that underlie the results reported in this paper (text, tables, figures and appendices) and the study protocol will be shared if requested. Data will be available beginning 12 months and ending 5 years following publication of this paper. Data will be available for researchers who provide a methodologically sound scientific proposal, which has been approved by an ethical committee. Proof of the latter should be provided. Analyses should achieve the aims as reported in the approved proposal. Proposals for data should be directed to j.rutges@erasmusmc.nl.
